# Mesmerism

**Published:** 1845-04

**Authors:** 


### MESMERISM.

Since the explosion of Homœopathy and Hydropathy, Mesmerism has been revived in Europe, and quite a sensation has recently been created on the subject by the celebrated Harriet Martineau. This lady enjoys an enviable literary reputation, and, although somewha older than unmarried ladies affect to be, still ranks with the Misses. Such an one, of all the world, is most likely to be afflicted with "nervous complaints," and Miss Martineau has sadly realized this in her own not remarkably feminine person. It appears from statements published by herself and her relative and former medical attendant, Mr. Greenhow, of New Castle, that she has been labouring under retroversion of the uterus, with dyspepsia and neuralgic pains of various parts, especially about the pelvis, which for a considerable time confined her to her chamber, and even to her bed and sofa, but that under the judicious advice of Mr. Greenhow and Sir Charles M. Clark, she had so far recovered as to be able to take some exercise, and otherwise afford hopes of a speedy recovery. It was at this juncture that a Mesmerist was allowed to minister to her case. Convalescence had already commenced, and needed only some strong mental co-operation to render it complete; and this the new excitement was well calculated to supply; accordingly she soon began to go abroad, take long walks, enjoy books and society, and, in short, to resume her accustomed habits. This, of course, has been seized on by the Mesmerists as a triumph over medicine, and the name of Harriet Martineau has become as familiar to that class of jugglers as it erst was to the circles of polite literature. Nor has this prostitution of her name and influence been wholly without her consent, and therefore she has no just ground of complaint, if, forgetting the natural delicacy of her sex in the course she has pursued, others, in commenting on her case, should forget it also.

In referring to this matter, the Dublin Medical Press has the following just remarks on what may not improperly be called *the spirit of the age.*

"But the fact is, that we are every day approaching nearer and nearer to the springs and sources of quackery in all its ramifications. Vanity, sheer vanity, egotism, and craving after notoriety, is more at the bottom of it than people think. Your consequential, ostentatious candidate for the attentions of the circle in which he moves, delights to assert his importance, and display his judgment by joining in the discovery of a cure, or the patronizing of a charlatan; and the restless spoiled child of indulgence has full scope for the gratification of caprices, in flying from one quack to another in pursuit of relief from real or imaginary ailments. This poor lady has been so completely inflated by the breath of flattery, and so thoroughly persuaded into a belief of her being a most wonderful personage, that she thinks it quite a duty to tell the world how she feels, and how matters go on in her inside. So every fussy valetudinarian gratifies his self-love by recounting his deeds of doctoring, and boasting of his sagacity and discrimination in the discovery of a miraculous cure; while at the same time he enjoys the satisfaction of displaying his ability to incur expense and to command resources and luxuries beyond the reach of less favoured mortals. In fact, this species of quack-fancier, whether male or female, is the same creature that we find flying about after all other kinds of novelties, and seeking by change of scene and new excitements to sustain the artificial condition of mind and body which he finds essential to his existence."

